# The Schizophrenia-Associated Kv11.1-3.1 Isoform Results in Reduced Current Accumulation during Repetitive Brief Depolarizations

**DOI:** 10.1371/journal.pone.0045624

**Published:** 2012-09-24

**Authors:** Juliane Heide, Stefan A. Mann, Jamie I. Vandenberg

**Affiliations:** 1 Victor Chang Cardiac Research Institute, Darlinghurst, NSW, Australia; 2 St. Vincent’s Clinical School, University of New South Wales, Darlinghurst, NSW, Australia; 3 Schizophrenia Research Institute, Darlinghurst, NSW, Australia; University of Bristol, United Kingdom

## Abstract

Recent genome wide association studies identified a brain and primate specific isoform of a voltage-gated potassium channel, referred to as Kv11.1-3.1, which is significantly associated with schizophrenia. The 3.1 isoform replaces the first 102 amino acids of the most abundant isoform (referred to as Kv11.1-1A) with six unique amino acids. Here we show that the Kv11.1-3.1 isoform has faster rates of channel deactivation but a slowing of the rates of inactivation compared to the Kv11.1-1A isoform. The Kv11.1-3.1 isoform also has a significant depolarizing shift in the voltage-dependence of steady-state inactivation. The consequence of the altered gating kinetics is that there is lower current accumulation for Kv11.1-3.1 expressing cells during repetitive action potential firing compared to Kv11.1-1A expressing cells, which in turn will result in longer lasting trains of action potentials. Increased expression of Kv11.1-3.1 channels in the brain of schizophrenia patients might therefore contribute to disorganized neuronal firing.

## Introduction

Schizophrenia is a severe mental disorder that affects between 0.5% and 1% of the population [1]. It is associated with a myriad of symptoms including psychosis, motivational impairment, affective dysregulation as well as cognitive abnormalities [2], [3]. The complexity and variability of the disease makes patient management very difficult. The causes of schizophrenia remain to be determined, however there is clearly a significant genetic contribution [4]. The gene candidates that have been identified to date [5] account for only a very small proportion of the known genetic contribution [6]. Consequently, there are significant ongoing efforts to identify new genes and to explore the possibility that the genes identified to date may open up novel targets for therapy.

In a recent study, single nucleotide polymorphisms (SNPs) in the second intron of the *KCNH2* gene on chromosome 7q36.1 were shown to be significantly associated with an increased risk for development of schizophrenia [7]. The SNPs were replicated in a separate Caucasian case control population [7] and confirmed in Turkish [8] and Japanese [9] population studies. *KCNH2* encodes for Kv11.1, a voltage-gated potassium channel, previously referred to as human *ether-à-go-go*-related gene (hERG). Kv11.1 channels have been extensively characterized, as they play a central role in repolarization of the cardiac action potential. Huffaker *et al.* showed that the SNPs, identified in the second intron of *KCNH2* gene, promote transcription from an alternative transcription start site and the expression of a primate and brain specific Kv11.1 potassium channel isoform referred to as KCNH2-3.1 or Kv11.1-3.1. In the 3.1 isoform the first 102 amino acids of the full length Kv11.1-1A isoform are replaced with six unique amino acids [7].

Kv11.1 channels have unusual gating properties, most notably slow activation and deactivation kinetics but very rapid and voltage dependent inactivation and recovery from inactivation kinetics [10]. The amino-terminal region of Kv11.1 is crucial for determining the slow deactivation kinetics of the channel [11]–[13]. Unsurprisingly then, the 3.1 isoform shows faster deactivation than the full length Kv11.1-1A isoform. However, to appreciate the differences in the effect that the 3.1 and 1A isoforms will have on the electrical properties of neurons we also need to know how the N-terminal truncation affects activation, inactivation and recovery from inactivation as well as deactivation.

In this study we show, using the whole-cell voltage-clamp technique, that in addition to faster deactivation kinetics, Kv11.1-3.1 channels have a significant depolarizing shift in steady-state inactivation compared to Kv11.1-1A with heterotetrameric Kv11.1-1A/Kv11.1-3.1 channels having an intermediate phenotype. The altered gating of the Kv11.1-3.1 channels has minimal effect on single short depolarization pulses, however, it results in substantially less accumulation of current during repetitive stimuli, which would result in adaptation of action potential firing rates in response to prolonged stimuli. Thus expression of the Kv11.1-3.1 isoform will result in little difference in action potential firing patterns at rest but will have significant effects during repetitive firing.

## Materials and Methods

### Molecular Biology

The C-terminal HA tagged Kv11.1-3.1 construct was generated using polymerase chain reaction. An oligonucleotide primer with the 3.1 specific sequence and an EcoRV restriction site was used as the 5′ primer and a primer covering the BstEII restriction site in *KCNH2* was used as the 3′ primer (F: 5′ AAGGGAGATATCGATCATCGCCGCCACCATGTCCTCC-CACTCTGCAGGGAGCTGCTTCCTATGTCTGGTGGATGTGG, R: 5′ ACAGGACCTT-GGGTGACCTTCTCAGTGACATTGTGGGTT). The product was digested with EcoRV and BstEII, ligated into *KCNH2* cDNA and subcloned into pIRES-neo and pIRES2-eGFP (Clontech laboratories, Mountain View, CA) vector. The Kv11.1-3.1 constructs were confirmed by bi-directional sequencing. Chinese hamster ovary (CHO) cells stably transfected with the Kv11.1-3.1 pIRES-neo construct were generated by transfecting CHO cells with linearized plasmid and selection in medium containing 1 mg/mL of G418 sulphate (Sigma-Aldrich, Australia). Expression of Kv11.1-3.1 in individual clones was verified by western blot. Stable cells were maintained in media containing 0.5 mg/mL of G418. For cells expressing the heterotetrameric Kv11.1-1A/Kv11.1-3.1 channels, Kv11.1-3.1 in pIRES2-eGPF was transfected onto CHO cells stably expressing Kv11.1-1A [14]. CHO cells were cultured in Dulbecco’s modified Eagle’s medium/F-12 (Invitrogen, Australia), supplemented with 10% fetal bovine serum (Sigma-Aldrich, Australia), 1x non-essential amino acids (Invitrogen, Australia), 1x GlutaMAX (Invitrogen, Australia) and maintained at 37°C in 5% CO_2_.

### Electrophysiology

Electrophysiological studies reported in the main manuscript were performed at 37°C. Additional experiments recorded at room temperature are described in the supporting information (Results S1, Figures S1-S4). Cells were trypsinized and plated onto glass cover slips. Glass capillary patch electrodes, with resistances of 2–4 MΩ when filled with internal solution, were made using a vertical two-stage puller (PP-830, Narishige, Tokyo, Japan). The internal solution contained (in mM): 120 potassium gluconate, 5 EGTA, 10 HEPES, 20 KCl, 1.5 Mg-ATP, pH 7.3 with KOH. Cells were superfused with external solution containing (in mM): 1 MgCl_2_, 1 CaCl_2_, 10 HEPES, 12.5 Glucose, 5 KCl, 130 NaCl, 0.1% dimethyl sulfoxide (DMSO), pH 7.4 with NaOH. The calculated junction potential for these solutions was −15 mV, which was corrected for in all experiments. Cells were voltage clamped in whole cell mode using an Axopatch 200B amplifier (Molecular Devices, Sunnyvale, CA). Current signals were digitized at 5 kHz and filtered at 1 kHz (except for inactivation protocols, which were digitized at 10 kHz and the short depolarization pulses, which were digitized at 100 kHz, and filtered at 5 kHz) and stored on an IBM-compatible PC interfaced with a Digidata 1440A analog-digital converter (Molecular Devices, Sunnyvale, CA). Series resistance was compensated by at least 80% in all experiments.

### Voltage Protocols

All voltages protocols used are illustrated on top of each panel. Rates of activation were measured using an envelope-of-tail protocol [15]. Cells were depolarized from a holding potential of −80 mV to 0 mV for variable durations before stepping to −120 mV where tail currents were recorded. To measure rates of deactivation, cells were first depolarized to +20 mV for 500 ms to fully activate the channels. Cells were then repolarized to voltages in the range −60 to −130 mV for 5 s and exponential functions fitted to the decaying portion of the current traces. After each sweep, cells were hyperpolarized to −120 mV for 100 ms to ensure full channel deactivation before reverting to the holding potential of −80 mV. This protocol was also used to measure the rates of recovery from inactivation although the voltage range was extended to voltages in the range −10 to −130 mV. Rates of inactivation were measured during a triple pulse protocol [15]. Cells were depolarized from a holding potential of −80 mV to +40 mV for 500 ms, repolarized to −80 mV for 10 ms, followed by voltage steps to membrane potentials in the range of +60 to −30 mV.

### Voltage-dependence of Steady-state Activation

Voltage-dependence of steady-state activation was measured using an isochronal tail current protocol [16]. Cells were depolarized from a holding potential of −80 mV, to voltages in the range of −100 to +40 mV for 4 s before stepping to −60 mV to record tail currents. Between sweeps, cells were held at −120 mV for 3 s to fully deactivate all channels. Steady-state activation curves were fitted with a modified Boltzmann equation:
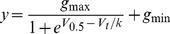
(1)where *V_0.5_* is the half-activation voltage, *V_t_* is the test potential, *k* is the slope factor, g_max_ is maximum conductance and g_min_ is the minimum conductance.

### Voltage-dependence of Steady-state Inactivation

Several different methods to measure steady-state inactivation have been described in the literature [17]. Most methods rely on a two pulse protocol and extrapolation of a deactivating Kv11.1 current back to the time of the voltage step. These methods work well for the 1A isoform of Kv11.1, but due to the much faster deactivation of the Kv11.1-3.1 isoform, these extrapolation methods do not provide very accurate estimates for Kv11.1-3.1. We therefore chose to use a method that relies on measuring the voltage dependence of the rates of inactivation and recovery from inactivation, and from these calculate the equilibrium constant for inactivation [18]. Specifically, a plot of the measured rates of inactivation and recovery from inactivation versus voltage gives rise to a chevron plot, which can be fitted with the equation:
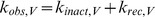
(2)where *k*_obs,v_ is the measured rate constant at any given voltage, *k*_inact,V_ and *k*_rec,v_ are the unidirectional forward and reverse rate constants at that voltage. The *k*_inact,V_ and *k*_rec,v_ values are then used to calculate the voltage dependent equilibrium constant for inactivation:
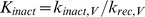
(3)with the midpoint for the voltage-dependence of steady-state inactivation occurring at the voltage where 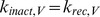.

### Data Analysis

Initial data analysis was performed using Clampfit 10.2 software (Molecular Devices, Sunny Vale, CA). All summary data are presented as mean ± SEM. Statistical comparisons (performed using ANOVA, followed by Tukey’s t-test) were carried out in Prism (GraphPad 5.04, La Jolla, CA,). A *P* value of <0.05 was considered significant.

## Results

Kv11.1 channels can exist in three different conformational states: closed, open and inactivated. The steady-state distribution and rates of interconversion between these three states were measured using the whole cell voltage clamp technique. We undertook a detailed analysis of the kinetics of the Kv11.1-3.1 and Kv11.1-1A isoforms at 37°C.

**Figure 1:**
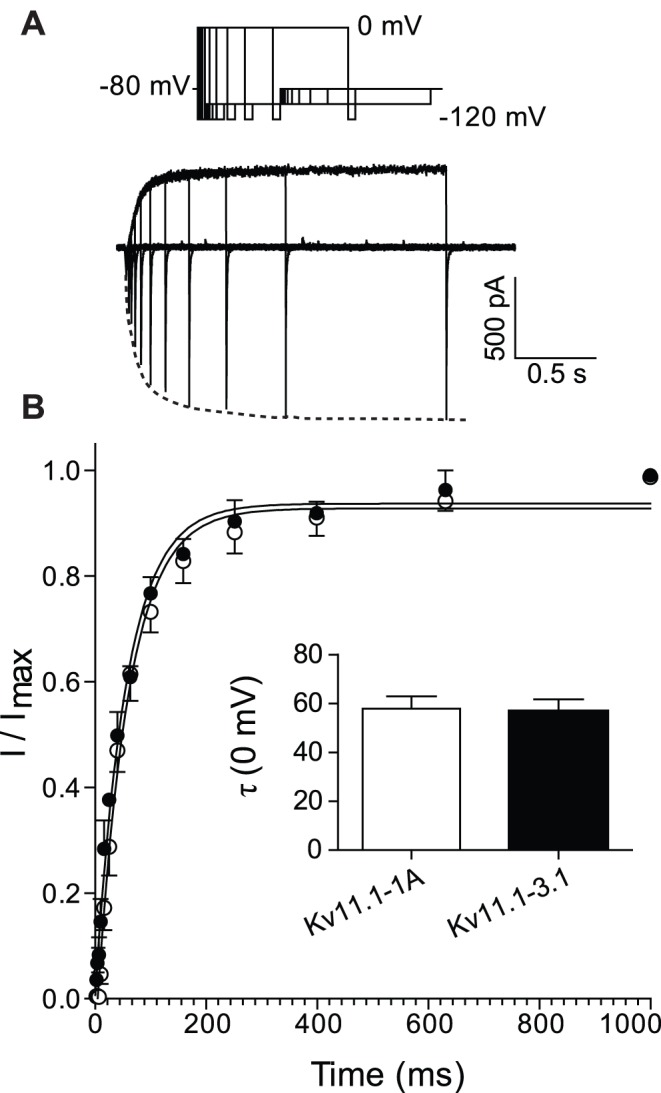
Rates of activation for Kv11.1 channels at 0 mV. A. Typical examples of Kv11.1-3.1 currents recorded at 37°C during an envelope-of-tails voltage clamp protocol to measure rates of activation at 0 mV. The voltage protocol is shown at the top of the panel. The dashed line highlights the peak tail current for each current trace. B. Normalized peak tail current plotted against duration of the test pulse for Kv11.1-1A (○) and Kv11.1-3.1 (•). The inset shows the mean ± SEM for time constants of activation (n = 4−5). τ_act, 0 mV_ for Kv11.1-1A (58±5 ms, n = 5) was not significantly larger than that for Kv11.1-3.1 (57±5 ms, n = 4).

Typical examples of a family of Kv11.1-3.1 currents recorded at 37°C during an envelope-of-tails protocol to measure the rate of activation at 0 mV are shown in Figure 1. The time constant of activation for Kv11.1-1A (58±5 ms, n = 5) was statistically indistinguishable from that for Kv11.1-3.1 (57±5 ms, n = 4) (Fig 1B). When rates of activation were measured at room temperature the small acceleration in the rate of activation for Kv11.1-3.1 compared to Kv11.1-1A became statistically significant (see Fig. S1). In addition, the Kv11.1-1A/Kv11.1-3.1 heterotetramer had rates of activation that were intermediate between those of the homotetrameric Kv11.1-1A and Kv11.1-3.1 channels (see Fig. S1 and Table S1).

**Figure 2:**
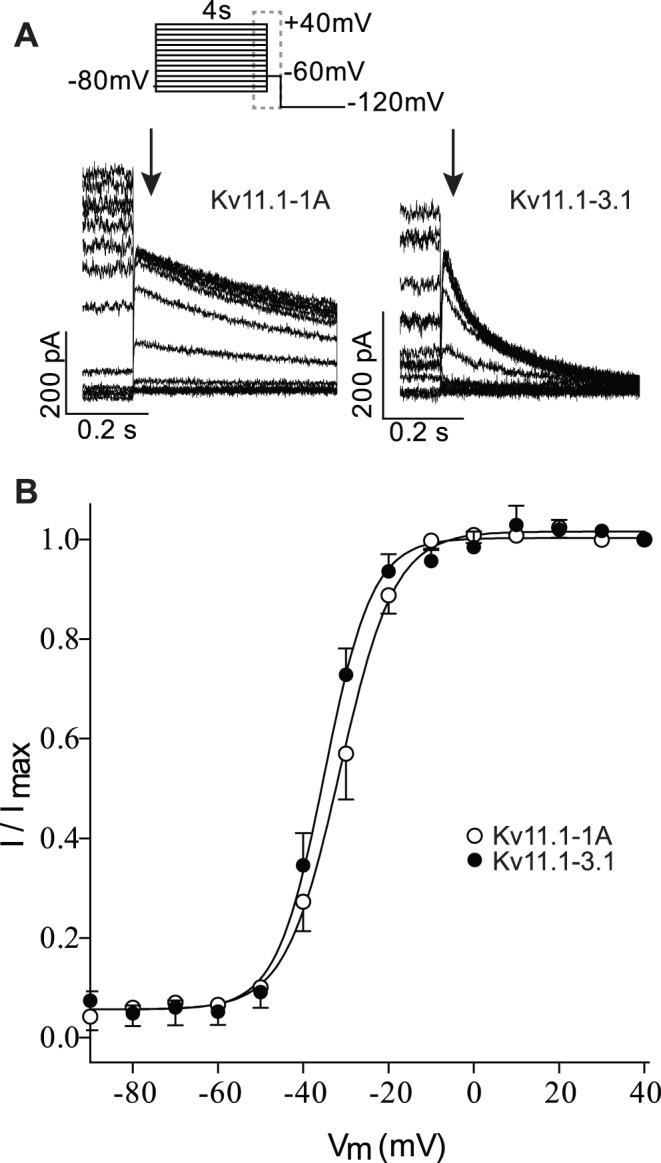
Voltage dependence of steady-state activation for Kv11.1-1A and Kv11.1-3.1. A. Typical families of current traces recorded at 37°C from Kv11.1-1A (left) and Kv11.1-3.1 (right) showing the last 100–150 ms of the activating step and 500 ms of the tail current recorded at −60 mV. Arrow indicates position where peak tail current was recorded. Inset at top of panel shows voltage protocol used to measure steady-state activation. B. Normalized peak tail currents plotted against voltages of the preceding test pulse for Kv11.1-1A (○) and Kv11.1-3.1 (•). Solid lines are fits of the Boltzmann function (see Eq. 1) giving V_0.5_ for steady-state activation of −31.4±1 mV for Kv11.1-1A and −35±1 mV for Kv11.1-3.1 (P<0.05).

We next investigated the steady-state activation properties of Kv11.1-1A and Kv11.1-3.1 channels. Typical families of currents recorded during 4 s depolarization steps to voltages in the range −90 mV to +40 mV are shown in Fig. 2A. The lines of best fit shown in Fig. 2B are Boltzmann functions (Eq. 1 in material and methods). Although small, the difference in the V_0.5_ of steady-state activation between Kv11.1-1A (−31±1 mV, n = 4) and Kv11.1-3.1 (−35±1, n = 5) was statistically significantly (p<0.05). This small difference between Kv11.1-1A and Kv11.1-3.1 was preserved at room temperature, with Kv11.1-1A/Kv11.1-3.1 having an intermediate phenotype (see Fig. S2 and Table S5).

**Figure 3:**
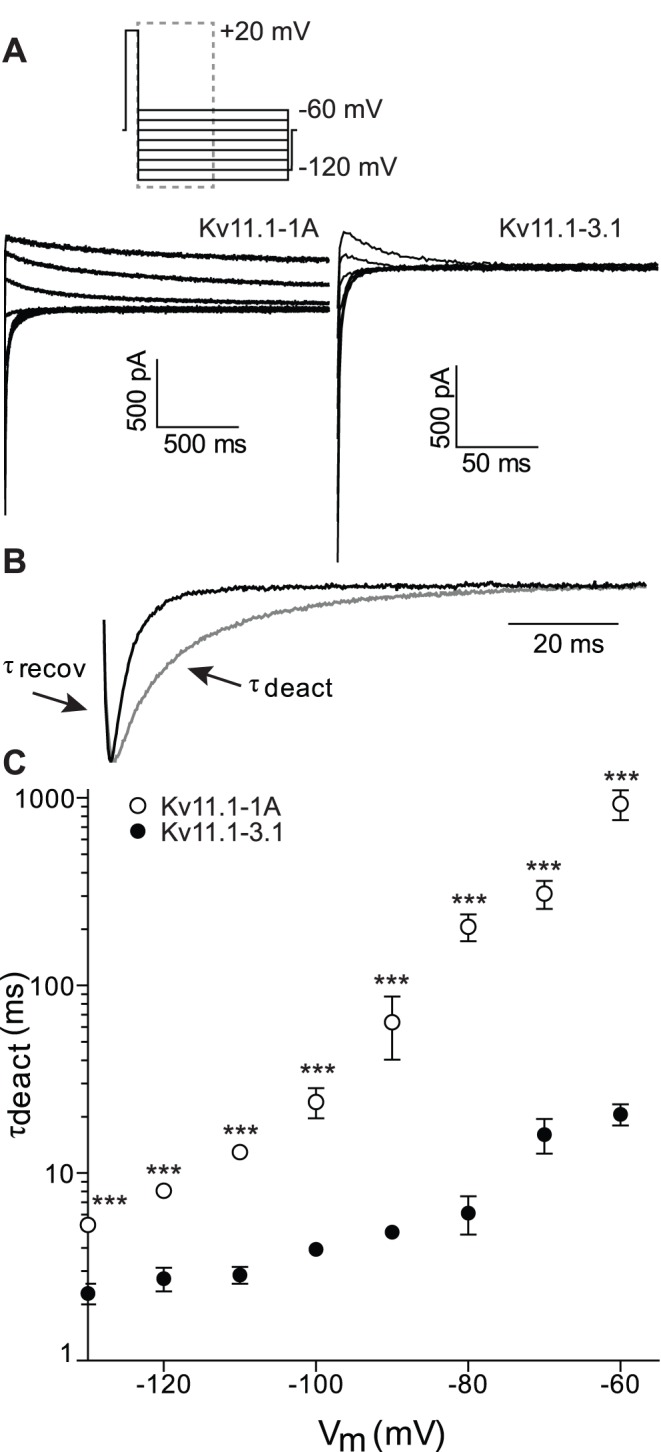
Rates of deactivation for Kv11.1-1Aand Kv11.1-3.1A. A. Typical currents recorded at 37°C during a protocol to measure rates of deactivation (voltage protocol show at top of panel, dashed box indicates the part of the voltage protocol for which current traces are shown) for Kv11.1-1A (left) and Kv11.1-3.1 (right). B. Magnification of the first 100 ms of the −120 mV tail current for Kv11.1-1A (grey) and Kv11.1-3.1 (black) show the characteristic hooked appearance reflecting recovery from inactivation followed by deactivation. C. Summary of τ_deact_ (mean ± SEM) over the voltage range of −130 mV to −60 mV for Kv11.1-1A (○) and Kv11.1-3.1 (•). ***: p<0.001.

It is apparent from the current traces shown in Fig. 2A that the rates of deactivation for Kv11.1-3.1 channels are markedly faster than for Kv11.1-1A. To further investigate the deactivation phenotype of the Kv11.1-3.1 channels, we measured rates of deactivation over the voltage range −130 to −60 mV (Fig. 3A). A magnification of the tail currents recorded at −120 mV, with the traces normalized to the maximum inward current, are shown in Fig. 3B. This clearly highlights the faster deactivation of Kv11.1-3.1 compared to Kv11.1-1A. Both currents show the characteristic hooked appearance reflecting recovery from inactivation followed by deactivation, which has been described extensively in the existing Kv11.1 literature [17]. Kv11.1-3.1 deactivates significantly faster (p<0.01) than Kv11.1-1A over the entire voltage range tested. For example, at −120 mV τ_deact_ was 3±0.4 ms for Kv11.1-3.1 compared to 8±0.5 ms for Kv11.1-1A (p<0.001). Similarly, at room temperature, Kv11.1-3.1 deactivates significant faster than Kv11.1-1A with Kv11.1-1A/Kv11.1-3.1 having an intermediate phenotype (see Fig. S3 and Table S2).

**Figure 4:**
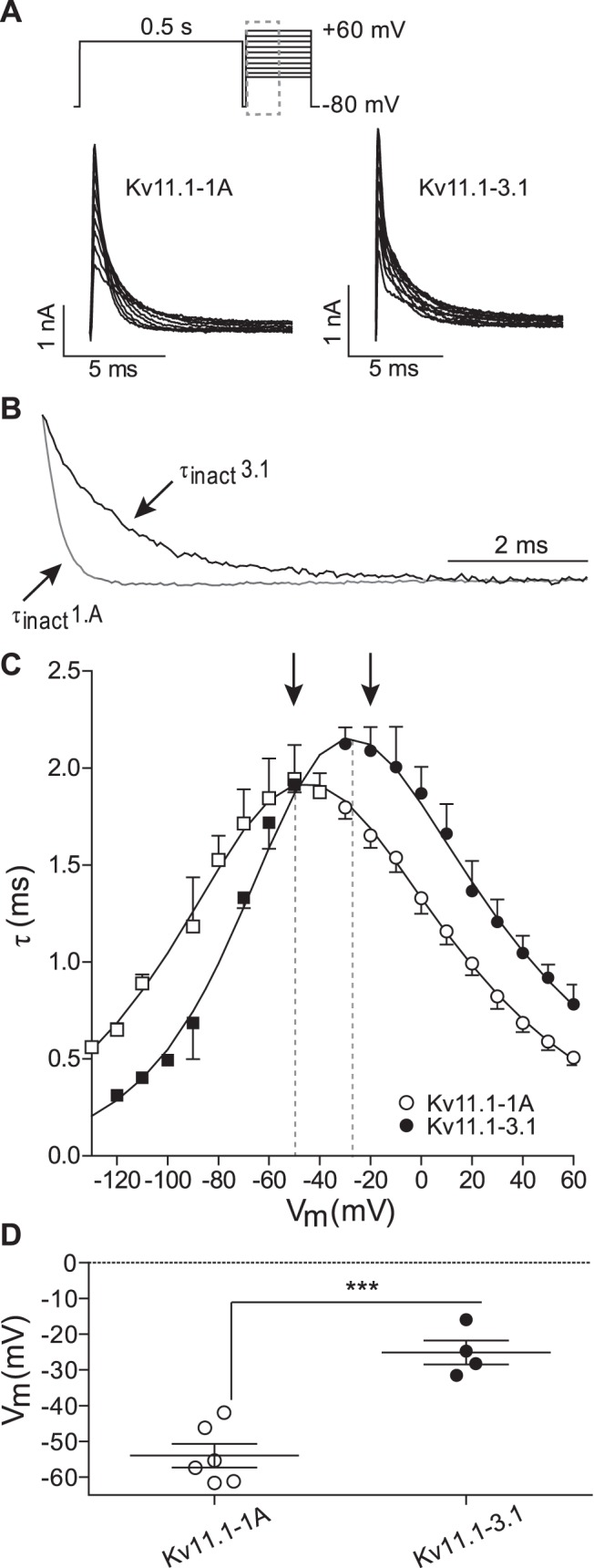
Inactivation properties of Kv11.1-1A and Kv11.1-3.1. A. Typical family of Kv11.1-1A (left) and Kv11.1-3.1 (right) current traces recorded at 37°C during a protocol to measure rates of inactivation (voltage protocol shown at top of panel A, dashed box highlights the part of the traces shown). B. Magnification of the first 8 ms of the +60 mV step for Kv11.1-1A (grey) and Kv11.1-3.1 (black) indicating slower inactivation for Kv11.1-3.1 compared to Kv11.1-1A. C. Summary of rates of recovery from inactivation for Kv11.1-1A (□) and Kv11.1-3.1 (▪) (τ_recov_, measured from protocol shown in Fig. 3) over the voltage range −130 mV to −40 mV and rates of inactivation (τ_inact_) for Kv11.1-1A (○) and Kv11.1-3.1 (•) over the voltage range **−**30 mV to +60 mV. Solid lines are the best fits of equation 2 to the data (see materials and methods). D. Midpoint of stead-state inactivation for Kv11.1-1A (○) and Kv11.1-3.1 (•) measured as the voltage at which τ_inact_ = τ_recov_ (see methods for details). ***: p<0.001.

Figure 4A shows typical current traces for Kv11.-1A (left) and Kv11.1-3.1 (right) recorded during a voltage protocol to monitor the rates of inactivation at potentials between **−**30 and +60 mV. A magnification of the +10 mV step is shown in Fig. 4B, highlighting slower rates of inactivation for Kv11.1-3.1 compared to Kv11.1-1A. Kv11.1-3.1 channels inactivated more slowly than Kv11.1-1A channels at all voltages in this range (Fig. 4C). The rates of recovery from inactivation, in the voltage range of **−**130 to **−**10 mV (Fig. 4C), were measured from the protocol shown in Fig. 3A. Kv11.1-3.1 channels recover from inactivation more rapidly than Kv11.1-1A channels over the tested voltages. Furthermore, the values for the V_0.5_ of steady-state inactivation (Fig. 4D, measured using Eq. 3, see material and methods) for Kv11.1-1A (**−**54±3 mV) and Kv11.1-3.1 (**−**25±3 mV), were statistically significantly different to each other (p<0.001). This shift in voltage-dependence of inactivation was also seen at room temperature with the heterotetramer Kv11.1-1A/Kv11.1-3.1 having an intermediate phenotype (see Fig. S4 and Table S5).

In summary, Kv11.1-3.1 channels have similar rates of activation but slower rates of inactivation at potentials >**−**20 mV, compared to Kv11.1-1A channels. The rates of recovery from inactivation, at potentials <**−**60 mV, and the rates of deactivation are also significant faster for Kv11.1-3.1 compared to Kv11.1.-1A. The voltage-dependence of steady-state activation for Kv11.1-3.1 is shifted ∼**−**4 mV relative to Kv11.1-1A. Kv11.1-3.1 also shows a significant shift to more depolarized potentials for the voltage-dependence of steady-state inactivation. Full details of all parameters measured are shown in Table S1–S5.

**Figure 5:**
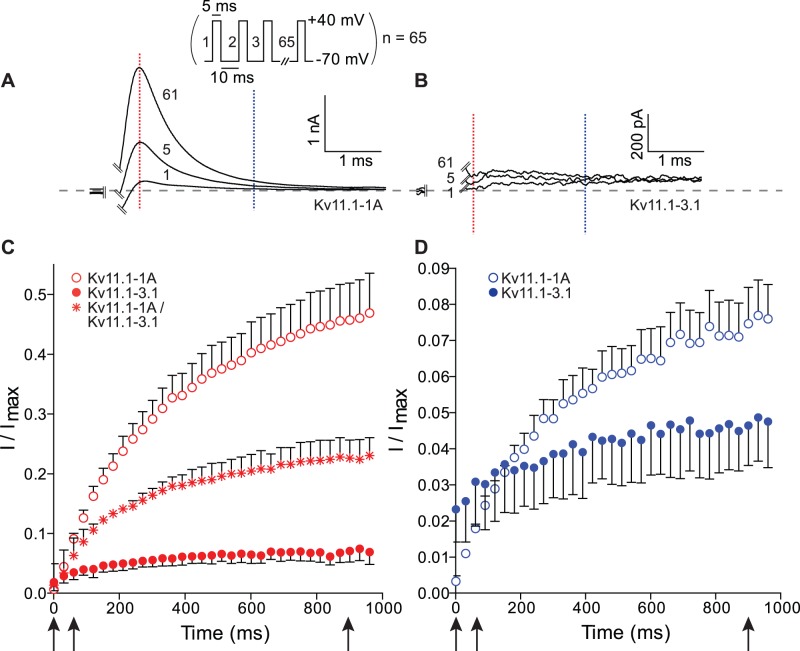
Action potential simulation for Kv11.1-A and Kv11.1-3.1. Typical current responses for the 1^st^, 5^th^ and 61^st^ pulse for A. Kv11.1-1A and B. Kv11.1-3.1 during a pulse protocol where cells were repetitively depolarized to +40 mV for 5 ms, from a holding potential of **−**70 mV with interpulse interval of 15 ms (voltage protocol shown at top of panel). Currents were measured at 1 ms (red dashed line) and 3 ms (blue dashed line) and the values normalized to the peak tail current recorded for each cell at **−**120 mV after a 1 s step to +40 mV to fully activate the channels (data not shown). C. Normalized currents measured at 1 ms (red symbols) plotted against time for Kv11.1-1A (○), Kv11.1-1A/Kv11.1-3.1 (*) and Kv11.1-3.1 (•) with the 1^st^, 5^th^ and 61^st^ pulse highlighted by the black arrows. D. Normalized currents measured at 3 ms (blue symbols) plotted against time for Kv11.1-1A (○) and Kv11.1-3.1 (•). 1^st^, 5^th^ and 61^th^ pulse highlighted with black arrows. The data for the Kv11.1-1A/Kv11.1-3.1 channels has been left out of panel D for purposes of clarity (due to overlapping error bars). Panel C and D only show every second data point for purposes of clarity.

To examine the physiological relevance of the difference in voltage-dependent gating between the Kv11.1-3.1 and Kv11.1-1A isoforms, we next looked at the current responses to repetitive depolarization pulses to mimic trains of neuronal action potentials. Figure 5 shows typical current responses for Kv11.1-1A and Kv11.1-3.1 for the 1^st^, 5^th^ and 61^st^ 5 ms depolarization pulse to +40 mV from a holding potential of **−**70 mV, recorded at 37°C. Normalized current responses measured 1 ms (shown in red) and 3 ms (shown in blue) into each depolarization pulse are shown in Figures 5C and 5D, respectively. When measured after 1 ms depolarization, the current size for Kv11.1-1A, Kv11.1-1A/Kv11.1-3.1 and Kv11.1-3.1 are similar for the 1^st^ pulse but thereafter the Kv11.1-1A currents increase to a much larger extent, plateauing at 0.49±0.02 for Kv11.1-1A compared to 0.23±0.01 for Kv11.1-1A/Kv11.1-3.1 and 0.07±0.003 for Kv11.1-3.1. When measured after 3 ms depolarization, the current magnitude for the Kv11.1-3.1 was significantly higher than for Kv11.1-1A on the 1^st^ pulse. However, the current magnitudes measured at 3 ms of each pulse reached similar levels after ∼10 pulses but thereafter the magnitude of the Kv11.1-1A currents increased to a larger extent reaching a plateau of 0.08±0.003 compared to 0.05±0.005 for Kv11.1-3.1 channels.

## Discussion

### The Kv11.1-3.1 Isoform has Multiple Effects on Channel Gating

In the Kv11.1-3.1 isoform the first 102 amino acids are replaced with six unique amino acids compared to the more abundant Kv11.1-1A isoform. The N-terminal region is well known to affect the deactivation kinetics of Kv11.1 channels and so it was expected that the Kv11.1-3.1 isoform should have faster kinetics. This was demonstrated by Huffaker *et al.* in the first report describing the 3.1 isoform [7] and confirmed in this study (see Fig. 3 and Fig. S3). The rates of activation (Fig. 1 and Fig. S1) and the voltage-dependence of steady-state activation (Fig. 2 and Fig. S2) were only minimally affected. Parameters for all gating variables are summarized in Table S1–S5.

Our data is consistent with numerous reports in the literature showing that various N-terminal truncations of the Kv11.1 channel selectively affect deactivation with no significant effects on rates of activation or steady-state activation [13], [19]. In addition to the major effect on deactivation, we also show that the Kv11.1-3.1 isoform has a major effect on the kinetics and voltage-dependence of steady-state inactivation (Fig. 4 and Fig. S4). Specifically, the rates of inactivation are slower at depolarized potentials (Fig. 4C) whereas the rates of recovery from inactivation are faster at hyperpolarized potentials (Fig. 4C). As a consequence, the mid-point for the voltage-dependence of steady-state inactivation is shifted in the depolarized direction for Kv11.1-3.1 compared to Kv11.1-1A channels, with the heterotetrameric channel having an intermediate phenotype (Fig. S4). Consequently, at modestly depolarized potentials the steady-state level of current flow though Kv11.1-3.1 channels will be significantly greater than in Kv11.1-1A channels. For example, at 0 mV only 5% of Kv11.1-1A channels will not be inactivated, whereas for Kv11.1-3.1 20% of the channels will not be inactivated. Thus, at depolarized potentials Kv11.1-3.1 channels will have a “gain of function” compared to Kv11.1-1A channels which is in marked contrast to the “loss of function” seen after depolarization steps caused by the faster deactivation of Kv11.1-3.1 channels [7].

### Physiological Implications of Altered Kv11.1 Gating

To investigate the physiological implication of the altered gating properties we investigated how the different isoforms would respond to repetitive short depolarization pulses in the voltage range typical of neuronal action potentials. Given that both isoforms have similar rates of activation and they both are largely inactivated at +40 mV, there was very little difference in the magnitude of the currents recorded during a single 5 ms depolarization step to +40 mV. However, due to the significant faster rates of deactivation (Fig. 3 and Fig. S3) and rates of recovery from inactivation (Fig 4 and Fig. S4), Kv11.1-3.1 channels almost fully close during the 10 ms repolarization step to −70 mV, while most Kv11.1-1A channels remain in the open conformation. Consequently, Kv11.1-1A currents accumulate to a much higher extent than Kv11.1-3.1. This is consistent with the data reported by Huffaker and colleagues [7]. Measuring the currents after 3 ms indicates that the first pulses for Kv11.1-3.1 start at a higher level compared to Kv11.1-1A. This can be explained by the significantly slower rate of inactivation for Kv11.1.-3.1 (Fig. 4 and Fig. S4) compared to Kv11.1-1A channels. Nevertheless, after repeated depolarization pulses the slower deactivation of Kv11.1-1A means that the current magnitude measured after 3 ms of the depolarization pulses still accumulates to a larger extent than for Kv11.1-3.1 channels. Thus overall, the faster deactivation kinetics of Kv11.1-3.1 is more important than the slower inactivation, resulting in less accumulation of Kv11.1-3.1 current compared to Kv11.1-1A current in response to multiple short depolarization pulses. Consequently, neurones containing Kv11.1-3.1 channels rather than Kv11.1-1A channels should permit more rapid and longer lasting trains of action potentials.

The reduced inactivation would be associated with an increased current flow during the depolarization phase of the action potential, which in turn would be expected to result in a shorter action potential. This however, is unlikely to be of any consequence for single action potentials in neuronal cells as the action potential is so short that the channels will not have activated to any significant degree.

### Relevance for Schizophrenia

The role of Kv11.1 channels in the cardiac repolarization is well studied [20] but the contribution of native Kv11.1 K^+^ current to the intrinsic electrical properties of CNS neurons is for the most part poorly understood [21]. Kv11.1-3.1 channels are primate and brain specific and have never been studied in their native environment. Both Kv11.1-1A and Kv11.1-3.1 isoforms are expressed at comparable levels in the hippocampus and the prefrontal cortex [7]. However, in patients with schizophrenia, positive for the M17, M30, M31 and M33 SNPs, the ratio of Kv11.1-3.1 to Kv11.1-1A expression in the hippocampus is ∼2.5-fold higher than in controls [7]. These changes in expression are very likely to influence the electrical properties of the cells as discussed above. However, before we can determine the overall significance of this altered cellular electrical phenotype we will need to define the precise cellular localizations of these channels and whether they are expressed in inhibitory neurons and/or excitatory neurons.

Despite intensive research, the pathobiology of schizophrenia remains obscure and consequently there is no cure for the disease [1], [22]. Therapeutics only rely on reduction of the symptoms. Without doubt, it is important to better understand the underlying mechanisms and how genes and their products impact brain function [23], [24]. Given that Kv11.1-3.1 is also expressed in healthy controls, it is likely that the higher disease incidence in patients with increased expression of Kv11.1-3.1 cannot be attributed to the altered levels of Kv11.1-3.1 alone but rather it is acting in combination with other risk factors (genetic or environmental) that remain to be determined.

## Supporting Information

Figure S1 **Rates of activation for Kv11.1-1A, Kv11.1-1A/Kv11.1-3.1 and Kv11.1-3.1 at 0 mV and +40 mV measured at room temperature.** Typical examples of Kv11.1-3.1 currents recorded at room temperature during an envelope-of-tails voltage clamp protocol to measure rates of activation at A. 0 mV and B. +40 mV. The voltage protocol is shown at the top of each panel. Dashed line highlights peak tail current for each current trace. C–D. Normalized peak tail currents plotted against duration of the test pulse for Kv11.1-1A (○), Kv11.1-1A/Kv11.1-3.1 (*) and Kv11.1-3.1 (•). Insets show the mean ± SEM for time constants of activation (n = 5−8). C. τ_act, 0 mV_ for Kv11.1-1A (662±70 ms, n = 8) was significantly larger than that for Kv11.1-3.1 (368±34 ms, n = 7, **: p<0.01) with Kv11.1-1A/Kv11.1-3.1 (475±72 ms, n = 5) not being significantly different to either Kv11.1-1A or Kv11.1-3.1 alone. D. τ_act, +40 mV_ for Kv11.1-1A (173±2 ms, n = 4) was significantly different than that for Kv11.1-3.1 (145±15 ms, n = 5, *: p<0.05).
(EPS)

Figure S2 **Voltage dependence of steady-state activation for Kv11.1-1A, Kv11.1-1A/Kv11.1-3.1 and Kv11.1-3.1 measured at room temperature.** A. Typical families of current traces recorded from Kv11.1-1A (left) and Kv11.1-3.1 (right) showing the last 200 ms of the activating step and 500 ms of the tail current recorded at −60 mV. Arrow indicates position where peak tail current was recorded. Inset at top of panel shows voltage protocol used to measure steady-state activation. B. Normalized peak tail currents plotted against voltages of the preceding test pulse for Kv11.1-1A (○), Kv11.1-1A/Kv11.1-3.1 (*) and Kv11.1-3.1 (•). Solid lines are fits of the Boltzmann function (see Eq. 1) giving V_0.5_ for steady-state activation of -22.7±1.4 mV for Kv11.1-1A, −25.3±0.8 mV for Kv11.1-1A/Kv11.1-3.1 and -27±0.9 mV for Kv11.1-3.1 (p<0.05 for Kv11.1-1A versus Kv11.1-3.1).
(EPS)

Figure S3 **Rates of deactivation for Kv11.1-1A, Kv11.1-1A/Kv11.1-3.1 and Kv11.1-3.1 measured at room temperature.** A. Typical tail currents recorded at -130 mV for Kv11.1-1A (grey) and Kv11.1-3.1 (black) following a 500 ms activation pulse to +20 mV (voltage protocol shown at top of the panel A). Tail currents show the characteristic hooked appearance reflecting recovery from inactivation followed by deactivation. B. Summary of τ_deact_ (mean ± SEM) over the voltage range of −130 mV to −60 mV for Kv11.1-1A (○), Kv11.1-1A/Kv11.1-3.1 (*) and Kv11.1-3.1 (•). ***: p<0.001 for Kv11.1-1A versus Kv11.1-3.1.
(EPS)

Figure S4 **Inactivation properties of Kv11.1-1A, Kv11.1-1A/Kv11.1-3.1 and Kv11.1-3.1A measured at room temperature.** A. Typical family of Kv11.1-1A (left) and Kv11.1-3.1 (right) current traces recorded during a protocol to measure rates of inactivation. Single exponential functions were fitted to the decaying portion of the tail currents for the voltages in the range 0 mV to +60 mV. B. Magnification of the +60 mV step for Kv11.1-1A (grey) and Kv11.1-3.1 (black) indicating slower inactivation for Kv11.1-3.1 compared to Kv11.1-1A. C. Summary of rates of recovery from inactivation (τ_recov_, measured from protocol shown in Fig. 3) over the voltage range −130 mV to −10 mV and rates of inactivation (τ_inact_) over the voltage range 0 mV to +60 mV for Kv11.1-1A (○), Kv11.1-1A/Kv11.1-3.1 (*) and Kv11.1-3.1 (•). Solid lines are the best fits of equation 2 to the data. D. Midpoint of steady-state inactivation measured as the voltage at which τ_inact_ = τ_recov_ (see methods for details). ***: p<0.001, *: p<0.05
(EPS)

Table S1 **Comparison of rates of activation at room temperature and 37°C.**
(DOCX)

Table S2 **Comparison of rates of deactivation at room temperature and 37°C.**
(DOCX)

Table S3 **Comparison of rates of inactivation at room temperature and 37°C.**
(DOCX)

Table S4 **Comparison of rates of recovery from inactivation at room temperature and 37°C.**
(DOCX)

Table S5 **Steady-state activation and inactivation for Kv11.1-1A, Kv11.1-1A/Kv11.1-3.1 and Kv11.1-3.1 at room temperature and 37°C**.
(DOCX)

Results S1 **In addition to characterising the channels at 37°C we performed a detailed analysis at room temperature.** In addition to the greater stability of the patch clamp recordings at room temperature the slowing of the kinetics at room temperature enabled us to investigate the subtle differences between Kv11.1-1A, Kv11.1-3.1, and the heterotetrameric Kv11.1-1A/Kv11.1-3.1 channels. In all cases the phenotype of the heterotetrameric Kv11.1-1A/Kv11.1-3.1 channels was intermediate between that of Kv11.1-1A alone and Kv11.1-3.1 alone, although in some cases the differences were not statistically significant. Rates of activation at 0 mV are shown in Fig. S1, steady-state activation in Fig. S2 and the rates of deactivation in Fig. S3. All the inactivation properties (rates of recovery from inactivation, rates of inactivation and the V_0.5_ of steady-state inactivation) are shown in Fig. S4. The data for all parameters at both room temperature and 37°C are also summarized in Tables S1-S5.
(DOCX)
