# Supplementary material for: The Schizophrenia-Associated Kv11.1-3.1 Isoform Results in Reduced Current Accumulation during Repetitive Brief Depolarizations
Source: PLoS One. 2012 Sep 24;7(9):e45624. doi: 10.1371/journal.pone.0045624 (PMC3454411; doi:10.1371/journal.pone.0045624)
Supplement: Table S1 — Comparison of rates of activation at room temperature and 37°C. (DOCX) [file pone.0045624.s005.docx]

**Table S1: Comparison of rates of activation at room temperature and 37°C.**

| **τ_act,0 mV_ (ms)** | **Kv11.1-1A** | **Kv11.1-3.1** | **Kv11.1-1A / Kv11.1-3.1** |
| --- | --- | --- | --- |
| **Room temp** | 453 ± 17.0^1^ | 303 ±17.0^2^ | 354 ± 54.0^3,4^ |
| **37°C** | 57.9 ± 5.1 | 56.7 ± 4.9^5^ | n/a |

^1^ P = 0.0079, F = 6.52, one way ANOVA;

^2^ p = 0.0043, paired t test (Kv11.1-1A and Kv11.1-3.1);

^3^ p = 0.10, paired t test (Kv11.1-1A and Kv11.1-1A / Kv11.1-3.1);

^4^ p = 0.13, paired t test (Kv11.1-3.1 and Kv11.1-1A / Kv11.1-3.1);

^5^ p = 0.41, paired t test (Kv11.1-1A and Kv11.1-3.1)
