# Supplementary material for: The Schizophrenia-Associated Kv11.1-3.1 Isoform Results in Reduced Current Accumulation during Repetitive Brief Depolarizations
Source: PLoS One. 2012 Sep 24;7(9):e45624. doi: 10.1371/journal.pone.0045624 (PMC3454411; doi:10.1371/journal.pone.0045624)
Supplement: Table S2 — Comparison of rates of deactivation at room temperature and 37°C. (DOCX) [file pone.0045624.s006.docx]

**Table S2: Comparison of rates of deactivation at room temperature and 37°C.**

| **τ_deact, -60 mV_ (ms)** | **Kv11.1-1A** | **Kv11.1-3.1** | **Kv11.1-1A / Kv11.1-3.1** |
| --- | --- | --- | --- |
| **Room temp** | 1849 ± 136.0^1^ | 89.0 ± 5.5^2^ | 336.0 ± 172.0^3,4^ |
| **37°C** | 930 ± 170 | 20.5 ± 2.7^5^ | n/a |
| **τ_deact, -90 mV_ (ms)** |  |  |  |
| **Room temp** | 391.0 ± 77.0^6^ | 26.1 ± 7.9^7^ | 144.0 ± 46.0^8,9^ |
| **37°C** | 63.8 ± 23.6 | 4.8 ± 0.4^10^ | n/a |
| **τ_deact, -120 mV_ (ms)** |  |  |  |
| **Room temp** | 60.5 ± 8.4^11^ | 12.2 ± 0.4^12^ | 35.0 ± 7.7^13,14^ |
| **37°C** | 8.1 ± 0.5 | 2.7 ± 0.4^15^ | n/a |

^1^ P = < 0.0001, F = 20.43, one way ANOVA;

^2^ p =0.00034, paired t test (Kv11.1-1A and Kv11.1-3.1);

^3^ p = 0.0061, paired t test (Kv11.1-1A and Kv11.1-1A / Kv11.1-3.1);

^4^ p = 0.05, paired t test (Kv11.1-3.1 and Kv11.1-1A / Kv11.1-3.1);

^5^ p = < 0.0001, paired t test (Kv11.1-1A and Kv11.1-3.1);

^6^ P = 0.0005, F = 13.34, one way ANOVA;

^7^ p = < 0.0001, paired t test (Kv11.1-1A and Kv11.1-3.1);

^8^ p = 0.00532, paired t test (Kv11.1-3.1 and Kv11.1-1A / Kv11.1-3.1);

^9^ p = 0.056, paired t test (Kv11.1-1A and Kv11.1-1A / Kv11.1-3.1);

^10^ p = 0.0369, paired t test (Kv11.1-1A and Kv11.1-3.1);

^11^ P = < 0.0001, F = 26.70, one way ANOVA;

^12^ p = < 0.0001, paired t test (Kv11.1-1A and Kv11.1-3.1);

^13^ p = 0.0028, paired t test (Kv11.1-3.1 and Kv11.1-1A / Kv11.1-3.1);

^14^ p = 0.059, paired t test (Kv11.1-1A and Kv11.1-1A / Kv11.1-3.1);

^15^ p = < 0.0001, paired t test (Kv11.1-1A and Kv11.1-3.1)
