# Supplementary material for: The Schizophrenia-Associated Kv11.1-3.1 Isoform Results in Reduced Current Accumulation during Repetitive Brief Depolarizations
Source: PLoS One. 2012 Sep 24;7(9):e45624. doi: 10.1371/journal.pone.0045624 (PMC3454411; doi:10.1371/journal.pone.0045624)
Supplement: Table S3 — Comparison of rates of inactivation at room temperature and 37°C. (DOCX) [file pone.0045624.s007.docx]

**Table S3: Comparison of rates of inactivation at room temperature and 37°C.**

| **τ_inact,-10 mV_** | **Kv11.1-1A** | **Kv11.1-3.1** | **Kv11.1-1A / Kv11.1-3.1** |
| --- | --- | --- | --- |
| **Room temp** | 8.9 ± 1.1^1^ | 11.4 ± 1.1^2^ | 11.5 ± 1.2^3,4^ |
| **37°C** | 1.5 ± 0.1 | 2.2 ± 0.2^5^ | n/a |
| **τ_inact, +40 mV_** |  |  |  |
| **Room temp** | 3.9 ± 0.2^6^ | 5.7 ± 0.4^7^ | 4.5 ± 0.4^8,9^ |
| **37°C** | 0.7 ± 0.1 | 1.1 ± 0.1^10^ | n/a |

^1^ P = 0.2032, F = 1.806, one way ANOVA;

^2^ p =0.1471, paired t test (Kv11.1-1A and Kv11.1-3.1);

^3^ p = 0.1374, paired t test (Kv11.1-1A and Kv11.1-1A / Kv11.1-3.1);

^4^ p = 0.9187, paired t test (Kv11.1-3.1 and Kv11.1-1A / Kv11.1-3.1);

^5^ p = < 0.0207, paired t test (Kv11.1-1A and Kv11.1-3.1);

^6^ P = 0.0036, F =8.358, one way ANOVA;

^7^ p = 0.00169, paired t test (Kv11.1-1A and Kv11.1-3.1);

^8^ p = 0.06374, paired t test (Kv11.1-3.1 and Kv11.1-1A / Kv11.1-3.1);

^9^ p = 0.1328, paired t test (Kv11.1-1A and Kv11.1-1A / Kv11.1-3.1);

^10^ p = 0.01467, paired t test (Kv11.1-1A and Kv11.1-3.1)
