# Supplementary material for: The Schizophrenia-Associated Kv11.1-3.1 Isoform Results in Reduced Current Accumulation during Repetitive Brief Depolarizations
Source: PLoS One. 2012 Sep 24;7(9):e45624. doi: 10.1371/journal.pone.0045624 (PMC3454411; doi:10.1371/journal.pone.0045624)
Supplement: Table S4 — Comparison of rates of recovery from inactivation at room temperature and 37°C. (DOCX) [file pone.0045624.s008.docx]

**Table S4: Comparison of rates of recovery from inactivation at room temperature and 37°C.**

| **τ_recov, -60 mV_ (ms)** | **Kv11.1-1A** | **Kv11.1-3.1** | **Kv11.1-1A / Kv11.1-3.1** |
| --- | --- | --- | --- |
| **Room temp** | 11.3 ± 0.5^1^ | 7.8 ± 0.5^2^ | 10.5 ± 1.3^3,4^ |
| **37°C** | 1.8 ± 0.1 | 1.7 ± 0.1^5^ | n/a |
| **τ_recov, -90 mV_ (ms)** |  |  |  |
| **Room temp** | 7.7 ± 0.6 | 4.3 ± 0.6^6^ | n/a |
| **37°C** | 1.2 ± 0.1 | 0.7 ± 0.1^7^ | n/a |
| **τ_recov, -120 mV_ (ms)** |  |  |  |
| **Room temp** | 4.2 ± 0.2^8^ | 2.2 ± 0.1^9^ | 3.4 ± 0.5^10,11^ |
| **37°C** | 0.7 ± 0.1 | 0.3 ± 0.1^12^ | n/a |

^1^ P = 0.0106, F = 6.245, one way ANOVA;

^2^ p =< 0.0001, paired t test (Kv11.1-1A and Kv11.1-3.1);

^3^ p = 0.5367, paired t test (Kv11.1-1A and Kv11.1-1A / Kv11.1-3.1);

^4^ p = 0.0658, paired t test (Kv11.1-3.1 and Kv11.1-1A / Kv11.1-3.1);

^5^ p = 0.7102, paired t test (Kv11.1-1A and Kv11.1-3.1);

^6^ p = 0.00357, paired t test (Kv11.1-1A and Kv11.1-3.1);

^7^ p = < 0.0001, paired t test (Kv11.1-1A and Kv11.1-3.1);

^8^ P =< 0.0001, F = 21.00, one way ANOVA,

^9^ p =< 0.0001, paired t test (Kv11.1-1A and Kv11.1-3.1),

^10^ p = 0.1268, paired t test (Kv11.1-1A and Kv11.1-1A / Kv11.1-3.1);

^11^ p = 0.00815, paired t test (Kv11.1-3.1 and Kv11.1-1A / Kv11.1-3.1);

^12^ p =< 0.0001, paired t test (Kv11.1-1A and Kv11.1-3.1)
