# Supplementary material for: The Schizophrenia-Associated Kv11.1-3.1 Isoform Results in Reduced Current Accumulation during Repetitive Brief Depolarizations
Source: PLoS One. 2012 Sep 24;7(9):e45624. doi: 10.1371/journal.pone.0045624 (PMC3454411; doi:10.1371/journal.pone.0045624)
Supplement: Table S5 — Steady-state activation and inactivation for Kv11.1-1A, Kv11.1-1A/Kv11.1-3.1 and Kv11.1-3.1 at room temperature and 37°C. (DOCX) [file pone.0045624.s009.docx]

**Table S5: Steady-state activation and inactivation for Kv11.1-1A, Kv11.1-1A / Kv11.1-3.1 and Kv11.1-3.1 at room temperature and 37°C**

| **Steady-state Activation** | **Kv11.1-1A** | | **Kv11.1-3.1** | | **Kv11.1-1A / Kv11.1-3.1** | |
| --- | --- | --- | --- | --- | --- | --- |
|  | **V_0.5_ (mV)** | **slope (mV)** | **V_0.5_ (mV)** | **slope (mV)** | **V_0.5_ (mV)** | **slope (mV)** |
| **Room temp.** | -22.7 ± 1.4^1^ | 10 ± 0.7 | -27 ± 0.9^2^ | 9.1 ± 0.8 | -25.3 ± 0.8^3,4^ | 11.2 ± 1.3 |
| **37°C** | -38.0 ± 0.6 | 4.9 ± 0.5 | -36.5 ± 0.4^5^ | 5.1 ± 0.4 | n/a | n/a |

| **Steady-state Inactivation** | **Kv11.1-1A V_0.5_ (mV)** | **Kv11.1-3.1 V_0.5_ (mV)** | **Kv11.1-1A / Kv11.1-3.1 V_0.5_ (mV)** |
| --- | --- | --- | --- |
| **Room temp** | -43.8 ± 2^6^ | -20.5 ± 2.4^7^ | -32.2 ± 2.9^8,9^ |
| **37°C** | -53.9 ± 3.3 | -25.2 ± 3.3^10^ | n/a |

Statistical values for V_0.5_ only:

^1^ P = 0.0311, F = 4.078, one way ANOVA;

^2^ p =0.0209, paired t test (Kv11.1-1A and Kv11.1-3.1);

^3^ p = 0.1385, paired t test (Kv11.1-1A and Kv11.1-1A / Kv11.1-3.1);

^4^ p = 0.1854, paired t test (Kv11.1-3.1 and Kv11.1-1A / Kv11.1-3.1);

^5^ p = 0.0279, paired t test (Kv11.1-1A and Kv11.1-3.1);

^6^ P =< 0.0001, F = 24.84, one way ANOVA;

^7^ p=< 0.0001, paired t test (Kv11.1-1A and Kv11.1-3.1);

^8^ p = 0.01118, paired t test (Kv11.1-1A and Kv11.1-1A / Kv11.1-3.1);

^9^ p = 0.01547, paired t test (Kv11.1-3.1 and Kv11.1-1A / Kv11.1-3.1);

^10^ p=< 0.0001, paired t test (Kv11.1-1A and Kv11.1-3.1)
