# Supplementary material for: The Schizophrenia-Associated Kv11.1-3.1 Isoform Results in Reduced Current Accumulation during Repetitive Brief Depolarizations
Source: PLoS One. 2012 Sep 24;7(9):e45624. doi: 10.1371/journal.pone.0045624 (PMC3454411; doi:10.1371/journal.pone.0045624)
Supplement: Results S1 — In addition to characterising the channels at 37°C we performed a detailed analysis at room temperature. In addition to the greater stability of the patch clamp recordings at room temperature the slowing of the kinetics at room temperature enabled us to investigate the subtle differences between Kv11.1-1A, Kv11.1-3.1, and the heterotetrameric Kv11.1-1A/Kv11.1-3.1 channels. In all cases the phenotype of the heterotetrameric Kv11.1-1A/Kv11.1-3.1 channels was intermediate between that of Kv11.1-1A alone and Kv11.1-3.1 alone, although in some cases the differences were not statistically significant. Rates of activation at 0 mV are shown in Fig. S1, steady-state activation in Fig. S2 and the rates of deactivation in Fig. S3. All the inactivation properties (rates of recovery from inactivation, rates of inactivation and the V0.5 of steady-state inactivation) are shown in Fig. S4. The data for all parameters at both room temperature and 37°C are also summarized in Tables S1-S5. (DOCX) [file pone.0045624.s010.docx]

**Results S1**

In addition to characterising the channels at 37ºC we performed a detailed analysis at room temperature. In addition to the greater stability of the patch clamp recordings at room temperature the slowing of the kinetics at room temperature enabled us to investigate the subtle differences between Kv11.1-1A, Kv11.1-3.1, and the heterotetrameric Kv11.1‑1A / Kv11.1-3.1 channels. In all cases the phenotype of the heterotetrameric Kv11.1‑1A / Kv11.1-3.1 channels was intermediate between that of Kv11.1-1A alone and Kv11.1-3.1 alone, although in some cases the differences were not statistically significant. Rates of activation at 0 mV are shown in Fig. S1, steady-state activation in Fig. S2 and the rates of deactivation in Fig. S3. All the inactivation properties (rates of recovery from inactivation, rates of inactivation and the V_0.5_ of steady-state inactivation) are shown in Fig. S4. The data for all parameters at both room temperature and 37ºC are also summarized in Tables S1-S5.
